# Mechanisms of Reciprocal Regulation of Gonadotropin-Releasing Hormone (GnRH)-Producing and Immune Systems: The Role of GnRH, Cytokines and Their Receptors in Early Ontogenesis in Normal and Pathological Conditions

**DOI:** 10.3390/ijms22010114

**Published:** 2020-12-24

**Authors:** Liudmila Zakharova, Viktoria Sharova, Marina Izvolskaia

**Affiliations:** Koltsov Institute of Developmental Biology, The Russian Academy of Sciences, 119334 Moscow, Russia; l-a-zakharova@mail.ru (L.Z.); izvolskaya@hotmail.com (M.I.)

**Keywords:** early ontogenesis, GnRH neuron migration, HPG and immune systems, thymic development, GnRH receptors, signal molecules, perinatal programming

## Abstract

Different aspects of the reciprocal regulatory influence on the development of gonadotropin-releasing hormone (GnRH)-producing- and immune systems in the perinatal ontogenesis and their functioning in adults in normal and pathological conditions are discussed. The influence of GnRH on the development of the immune system, on the one hand, and the influence of proinflammatory cytokines on the development of the hypothalamic-pituitary-gonadal system, on the other hand, and their functioning in adult offspring are analyzed. We have focused on the effects of GnRH on the formation and functional activity of the thymus, as the central organ of the immune system, in the perinatal period. The main mechanisms of reciprocal regulation of these systems are discussed. The reproductive health of an individual is programmed by the establishment and development of physiological systems during critical periods. Regulatory epigenetic mechanisms of development are not strictly genetically controlled. These processes are characterized by a high sensitivity to various regulatory factors, which provides possible corrections for disorders.

## 1. Introduction

It is currently believed that the reciprocal regulation of the hypothalamic-pituitary-gonadal (HPG) and immune systems is laid down already at the initial stages of their development and is controlled by neuropeptides, hormones, neurotransmitters, and immunomediators throughout the entire period of life [1,2,3,4,5,6,7]. The signals generated by the HPG axis, the main participants of which are gonadotropin-releasing hormone (GnRH), gonadotropins, and sex steroids [8], coordinate the development and functioning of the immune system, and immunomediators, in particular, cytokines and thymic peptides, influence the HPG axis [2]. The health of the adult offspring depends on how these interactions are formed and developed in the early, critical periods of ontogenesis, when the structure and functions of the HPG and immune systems are formed and epigenetic mechanisms are implemented that ensure their adaptive plasticity [2,9]. During early development, neurohormones, including GnRH, begin to be synthesized in fetal tissues before neural transmission is formed. They are found in the general blood circulation in high concentrations and carry out not only regulatory, but also morphogenetic functions [6,10,11,12]. During development, different signal molecules, specifically, serotonin and noradrenaline [13], semaphorins [14,15], gamma-aminobutyric acid (GABA) [16], involved in the regulation of the functions of the GnRH system of mature individuals are able to influence the migration of GnRH neurons, depending on their spatial or temporal location.

The initial activation of the HPG axis by GnRH occurs in utero, then in the early postnatal and pubertal periods. The initiation of puberty is associated with the activation of the HPG axis as a result of impulse secretion of GnRH in the hypothalamus [5,8,17]. Axons of GnRH neurons release GnRH into the portal system with a certain frequency, creating in it the concentration necessary to start secreting gonadotropins, which, in turn, stimulate the secretion of sex steroids. The key regulator of GnRH secretion is the KISS1 gene product kisspeptin (KISS1) and its receptor (KISS1R) [18]. The main form of the kisspeptin family, kisspeptin-54, contains a decapeptide that determines the functions of this family after binding to the specific receptor GPR54 (G protein receptor 54) or KISS1R [19,20,21].

**Figure 1:**
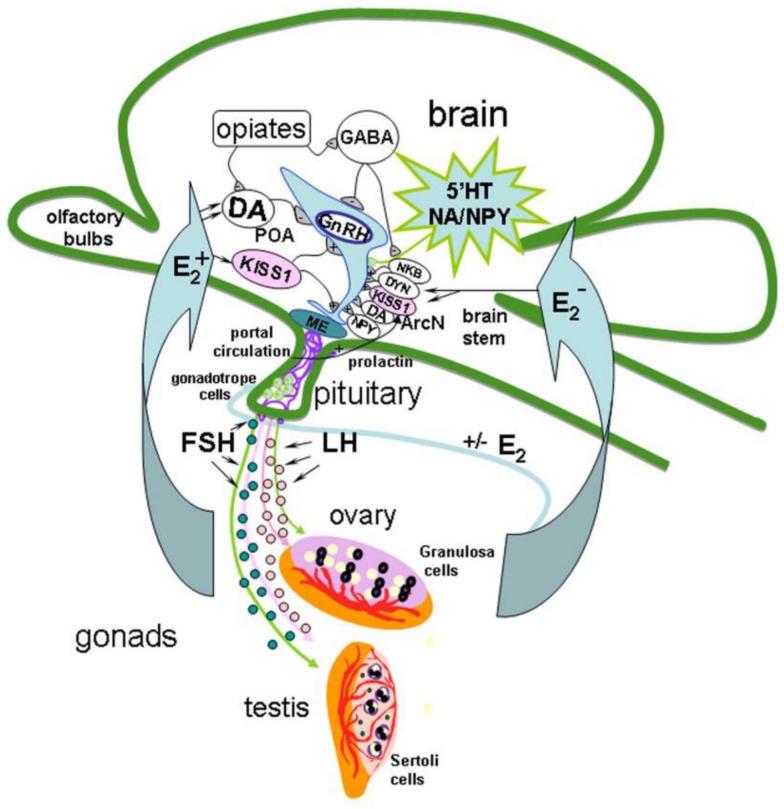
Model of the gonadotropin-releasing hormone (GnRH) neuron regulation in hypothalamic-pituitary-gonadal axis in adults. The mechanism of GnRH secretion regulation in the hypothalamus includes different signal molecules produced in different brain regions such as preoptic area (POA), locus coeruleus, raphe nucleus and brain stem, in particularly, serotonin (5′HT), dopamine (DA), noradrenaline (NA), gamma-aminobutyric acid (GABA), kisspeptin (KISS1), neuropeptide Y (NPY), opiates, neurokinin B (NKB) and dynorphin (DYN), which colocalize with KISS1 in the arcuate nucleus (ArcN), are also involved in the generation of GnRH impulses. NKB initiates the onset of GnRH impulse secretion in median eminence (ME), and DYN initiates its termination. In turn, axons of GnRH neurons release GnRH into the portal system in necessary concentration to trigger luteinizing hormone (LH) and follicle-stimulating hormone (FSH) secretion, which stimulate the secretion of sex steroids. Sex steroids, in turn, regulate the GnRH and LH synthesis in the brain and pituitary.

The mechanism of GnRH secretion regulation in the hypothalamus includes a network of various neurons, including KISS1-producing ones, which can act on GnRH neurons through separate or multiple neuronal systems [22,23]. GnRH- and KISS1 neurons are located in the same regions of the hypothalamus, and GnRH neurons express KISS1R [18,24]. Axons of KISS1 neurons form pericapillary plexuses at the site of GnRH secretion [25]. Neurokinin B (NKB) and dynorphin, which colocalize with KISS1 in the arcuate nucleus and are linked by axosomatic synapses, are also involved in the generation of GnRH impulses [26]. It is assumed that NKB initiates the onset of GnRH impulse secretion, while dynorphin initiates its termination [27]. General cues of GnRH neuron regulation in HPG axis is presented on Figure 1.

Along with the modulation of gonadotropins, GnRH is also involved in the modulation of sexual behavior, transmission of olfactory signals, and the formation and functioning of the immune system [2,28,29]. A variety of GnRH forms with different origins, structures, and functional significance have been revealed [29]. In all amniotes at least two GnRH forms are present (GnRH1 and GnRH2), which are encoded by separate genes and, despite their comparable cDNA and genomic structures, are clearly distinguished [30,31]. They mediate their effects through specific receptors associated with the transmembrane G protein. In humans the hypothalamic GnRH regulates gonadotropin secretion through the pituitary GnRH type I receptor via activation of G(q) [32]. In addition to the brain, expression of GnRHs and/or their receptors (GnRHRs) was also detected in the liver, heart, skeletal muscles, kidneys, mammary gland, testes, ovaries, prostate, endometrium, placenta during pregnancy, and in cells of immune system, in the splenocytes, thymocytes and peripheral blood T-cells, both CD4^+^ and CD8^+^ subsets, at different stages of ontogenesis [3,6,11,28,33,34].

The main source and target of circulating GnRH1 are the hypothalamus and the pituitary gland. Its functions are realized through endocrine mechanisms, while the functions of extracerebral GnRHs, which are synthesized locally, are apparently associated with autocrine and/or paracrine regulation [31,35]. In the thymus and spleen GnRH modulates the proliferation of various subpopulations of T lymphocytes, the activation of natural killer cells and the synthesis of cytokines [2,3,6].

In turn, immunomediators, in particular, cytokines, are modulators of the development of various brain structures, including the GnRH system, as well as modulators of steroidogenesis and gonadal functions in males and females [36,37]. In adults, cytokines are involved in maintaining homeostasis and regulating immune responses not only in the immune system at the periphery, but also in the brain. In the neurons and glial tissue of the brain, the expression of cytokines and their receptors, which are identical or closely related to the cytokines synthesized by the peripheral immune system, were detected. They affect the number and the so-called “immunocompetence” of nerve cells [38,39].

Bacterial or viral activation of the mother’s immune system during pregnancy leads to the development of systemic inflammation, characterized by an increase in the synthesis of proinflammatory cytokines, which have a negative effect on the development of the fetal brain [37,39,40,41,42,43,44,45,46,47]. The changes occurring in the brain lead to disorders in the development of the nervous and endocrine systems, including HPG, a decrease in reproductive capacity or infertility in adult offspring [40,46,48,49,50]. At present, a sufficient amount of data has appeared to prove that inflammation of the central nervous system (CNS) is associated with almost all neurological diseases that develop in remote periods of life [51,52,53,54,55]. Neuroinflammation can be generated by signals from cytokines and cells of the immune system migrating to the CNS from the periphery, as well as cytokines synthesized locally in the brain [47,48,49,50,51,52,53,54,55,56]. Cytokines can realize their effects both by direct influence on GnRH neurons through specific receptors, and indirectly, through the secretion of other mediators such as opioids, prostaglandins, catecholamines, GABA, glutamate, and nitric oxide (NO) [57,58,59].

The regulation of the processes of formation of physiological systems is characterized by lability and sensitivity to many regulatory factors. This opens up opportunities for correcting impairments of the development and functioning of the HPG system.

In our studies, we tried to reveal the role of GnRH in thymus development and possible mechanisms underlying the GnRH effects in early development, in particular, its influence on the cytokine expression in the fetal thymus in rodents, on the one hand, and the influence of cytokines on the development of the HPG system, on the other hand. In addition, long-term effects of prenatal GnRH deficiency on the functions of the immune system, and the influence of prenatal inflammation induced by bacterial lipopolysaccharide (LPS) on development and functioning of HPG system were studied. We are also searching approaches to reversing reproductive disorders induced by systemic inflammation in early ontogenesis.

This review article presents an analysis of our own and published data on possible mechanisms of regulation of the development and functioning of the GnRH-producing and immune systems in ontogenesis, the role of GnRH, cytokines and other signaling molecules, and their receptors in these processes in health and disease.

## 2. Effects of Different Signal Molecules on the Normal Development of the GnRH System

**Figure 2:**
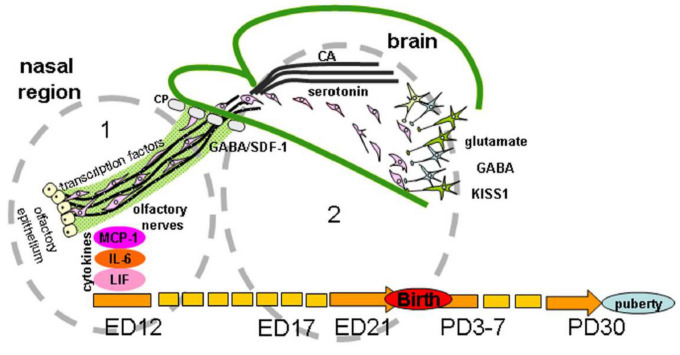
Regulation of gonadotropin-releasing hormone (GnRH) neuron migration in nasal region (1) and brain (2) during development. GnRH neurons originate outside the brain in nasal olfactory epithelium and later neurons migrate using the surface of olfactory/terminal/vomeronasal nerves through the cribriform plate of the ethmoid bone (CP) to the forebrain on embryonic days (ED) 11-15 in mice and ED 12-17 in rats. Their migration is orchestrated by different signal molecules, such as cell adhesion molecules (polysialylated neural cell adhesion molecules (NCAM) in rats or peripherin in mice), soluble guidance-cue factors (semaphorins, Slit proteins, netrins, reelin, stromal cell-derived factor 1 (SDF-1), etc.), cytokines (monocyte chemoattractant protein-1 (MCP-1), interleukin-6 (IL-6), leukemia inhibitory factor (LIF)), neurotransmitters (gamma-aminobutyric acid (GABA), glutamate, monoamines (serotonin, catecholamines (CA)), and transcription factors (Pax6, AP-2α, Gli3, Ebf2 Nhlh2 VAX1 Mash-1, Math4A, Math4/neurogenin1, NeuroD, Olf-1, GATA-4). In rats, GnRH-neurons reach their final position on ED19 and after the receiving of correct afferent innervation they are capable of performing main function—to regulate the reproductive axis in adults. The same signal molecules can perform different functions or other molecules can be included at different stages of the development of the GnRH system. For instance, NCAM apparently participate in laying the route for GnRH neurons and do not play a key role in their migration. Transcription factors are involved in the initial stages of the differentiation of olfactory placodes and the precursors of GnRH neurons, cytokines in the development of olfactory structures and in the migration of GnRH neurons and GnRH secretion. Neurotransmitters take part in the migration of GnRH neurons mainly at the stage of their entry into the forebrain. Guidance-cue molecules are involved in the development of the olfactory system and contribute to directing the migration of GnRH neurons in the nasal head region and forebrain. Kisspeptin (KISS1) signaling is well demonstrated as a key component for the onset of puberty, GnRH neurons express KISS1 in embryonic mice brain. Moreover, KISS1 may inhibit GnRH neuronal movement and plays a role of stop molecule for GnRH neuronal migration. Abbreviation: PD—postnatal day.

In vertebrates, most of the GnRH neurons are formed in the prenatal period outside the brain from the epithelium of the olfactory placodes and regulatory or morphogenetic factors are involved in the differentiation of these placodes [60]. Then GnRH neurons migrate to the forebrain, where they are also located in adults. Signaling molecules affecting the development of GnRH neurons are usually divided into groups according to functions closely related to the sites of origin, migration, and definitive location of neurons. At least five groups of signaling molecules are distinguished: (1) cell adhesion molecules, (2) soluble guidance-cue factors, (3) cytokines, (4) neurotransmitters, and (5) transcription factors (Figure 2).

As noted above, GnRH neurons migrating in the nasal head region move along the olfactory, terminal, and vomeronasal nerves. After entering the forebrain, these neurons migrate caudally along a temporary projection of the vomeronasal nerve to the septopreoptic area [61]. The general pattern of GnRH system development is similar in most mammals, although there are natural differences in the timing of neuron formation and migration depending on the duration of pregnancy and the relative degree of offspring development at birth in individual species.

The process of migration is conventionally divided into three stages: (1) intranasal migration, (2) passage through the cribriform plate of the ethmoid bone, and (3) intracerebral migration (Figure 2). In normal development, each stage is characterized by a unique set of factors such as cell adhesion proteins, gradients of guidance-cue molecules, and a specific cellular microenvironment producing neurotransmitters and neuromodulators necessary for a successful migration of GnRH neurons. For example, suppression of β1-integrin, which mediates the functions of cell adhesion proteins and guidance cues, impairs the migration of GnRH neurons in mouse fetuses, with a consequent delay in puberty and impairment of fertility in adult animals [62]. G-protein coupled receptors, in particular, prokineticin 2 (PROK2) and its receptor (PROKR2) were shown to modulate GnRH neuron migration. The mice null for PROKR2 had a loss of GnRH neurons in the forebrain on embryonic day 13.5 (ED13.5) and in adults, had formed a huge and tangled web of olfactory vomeronasal axons that would alter GnRH neuron movement [63].

### 2.1. Adhesion Molecules

In rats, GnRH neuron migration in the nasal head region is closely linked with fascicles of nerve fibers expressing polysialylated neural cell adhesion molecules (PSA-NCAM) [61]. Various experiments on the removal of NCAM from the migration route of GnRH neurons (using gene knockout, enzymatic treatment, or anti-NCAM antibodies) have shown that such interventions markedly reduce the number of migrating neurons but do not completely block their migration in rats [64,65,66]. Thus, NCAM apparently participate in laying the route for GnRH neurons but do not play a key role in their migration.

Moreover, it has been shown that these neurons in the nasal head region also migrate along nerve fibers expressing other cell adhesion proteins, namely, TAG-1 and CC-2 [61,67]. In mice, GnRH neurons appear to migrate along fascicles of nerve fibers expressing peripherin, an intermediate filament protein [68].

### 2.2. Guidance-Cue Molecules

It has been shown that a number of guidance-cue molecules participate in the development of the olfactory system. They include various chemoattractants, chemorepellents, and chemotrophic factors such as semaphorins, Slit proteins, netrins, reelin, etc. These factors guide the growth of olfactory nerve axons, and some of them supposedly contribute to directing the migration of GnRH neurons in the nasal head region and forebrain. The semaphorins play a critical role in GnRH neuron biology: on the one hand, regulating their migration and survival during embryonic development and, on the other hand, controlling the plasticity of the median eminence in terms of its response to varying sex steroid hormone levels [14,15,69,70]. For instance, SEMA 3A, SEMA 4D, SEMA 7A are responsible for vomeronasal axon guidance and GnRH neuron migration, SEMA 3E for neuron survival during the developmental period, and at the same time, SEMA 3A takes part in the median eminence plasticity and SEMA 7A in GnRH neuron plasticity in adults [71]. Mutations in 3 semaphorins (SEMA3A, SEMA3E, SEMA7A) and in their receptors (PLXNA1, NRP1, NRP2) have been found in isolated GnRH deficiency patients [15,71,72].

The secreted Slit glycoproteins (Slit2) and Roundabout receptors (Robo3) are also involved in the migration of GnRH neurons. Specifically, mice lacking Slit2 contained fewer GnRH neurons in the forebrain compared with wild-type controls, resulting in much reduced innervation of the median eminence [73,74]. Another glycoconjugate (glycosyltransferase β1,3 *N*-acetylglucosaminyltransferase-1 (β3GnT1) helps synthesize lactosamine with maximum expression on ED13. Mice with disrupted expression of β3GnT1 show GnRH neurons retained in the nasal area on ED15, fewer neurons in the forebrain and misdirection (those neurons are displaced in the dorsal rather than the ventral forebrain) [75]. Reelin is an extracellular glycoprotein and expressed by the vomeronasal neurons but not GnRH neurons. Reeler mice had a lower numbers of GnRH neurons in forebrain associated with further decreased fertility [76]. Reelin provides positional cues to radially migrating neurons in the cerebral cortex and plays a role in guiding the migration of GnRH neurons in the developing basal forebrain [76]. Netrins are a family of secreted proteins that are involved in the formation of many nerve tracts in the brain. The transmembrane protein DCC has been identified as a receptor for netrins. This protein is found in nerve fibers that express peripherin and serve as a guideway for GnRH neuron migration in the nasal head region [77]. The DCC mRNA has been detected in some GnRH neurons located in this region, but not in neurons that have already entered the forebrain [78].

Another chemoattractant protein playing such a role is the hepatocyte growth factor/scatter factor (HGF/SF), with c-Met tyrosine kinase receptors for this protein, being found in the immediate vicinity of migrating GnRH neurons [79]. It has been shown that the chemokine stromal cell-derived factor 1 (SDF-1) and one of its receptors, CXCR4, also participate in the intranasal migration of GnRH neurons [80,81]. SDF-1 appears to accelerate the speed of their movement via changes in potassium concentration inside the cells [16].

### 2.3. Cytokines

Cytokines, as a large and diverse family of regulators secreted by the glial cells of the nervous system and by numerous cells of the immune system, in particular, leukemia inhibitory factor (LIF), monocyte chemoattractant protein-1 (MCP-1), and interleukin (IL)-6, take part not only in GnRH secretion, but also in the development of olfactory structures and migration of GnRH neurons. During the period of intranasal migration of GnRH neurons to the brain, expression of these cytokines and their receptors is observed in this area [37,82]. Receptors for IL-6 were identified on the migration pathway GnRH neurons on the olfactory and vomeronasal nerves [37]. On the neuronal cells of the Gnv-4 line, which synthesize GnRH, receptors for the α chain of IL-6 are also expressed [60]. Using GN11 cells as a model of immature, migratory GnRH neurons, Magni et al. [83] showed that LIF induced their chemokinesis. MCP-1 and its receptor, chemokine (C-C motif) receptor-2 (CCR2), were found to be expressed in the hypothalamic GnRH neurons, as well as in GT1-7 and GN11 cell lines, with MCP-1 stimulating migration of these immature GnRH neurons [84].

Moreover, on the germ cells, including oocytes in primordial follicles, of human fetal ovaries receptors for IL-6 and LIF were identified, and their expression was increased significantly with increasing gestation [85]. On the cells of the urogenital tract, receptors for LIF were also detected [86]. An increase in the level of LIF in the blood of a pregnant female rat after its exogenous administration is accompanied by an increase in the level of this cytokine in the blood and cerebrospinal fluid of fetuses [87] and an increase in the level of MCP-1 in the mother was accompanied by its increase in the brain of fetuses [41]. IL-6 can be transported from the mother through the placental barrier to the fetus [88].

IL-1β has been shown to reduce the spontaneous expression of c-fos protein located within the nuclei of GnRH neurons in proestrus rats. The in vivo data demonstrate inhibition of GnRH, whereas in vitro studies demonstrated that IL-1β stimulates GnRH release [89].

### 2.4. Neurotransmitters

Neurotransmitters control the migration of GnRH neurons mainly at the stage of their entry into the forebrain. In mice, their migration is retarded at the cribriform plate, against the background of an increase in intracellular calcium resulting from GABA-induced tonic polarization of these cells [16,90]. This delay may be necessary for the maturation of GnRH neurons or reorganization of their behavior prior to entering the forebrain. GABA also has an effect on their distribution in the forebrain. Glutamate is another neurotransmitter influencing the migration of GnRH neurons, with its influence being different in the nasal head region and in the forebrain. In particular, treatment of pregnant mice with AMPA (α-amino-3-hydroxy-5-methyl-4-isoxazolepropionic acid) receptor blocker has proved to retard the entry of GnRH neurons into the forebrain in fetuses [91]. There have been shown that the spatiotemporal segregation of glutamic acid decarboxylase isoforms (GAD) defines distinct GABA signaling functions in the developing mouse olfactory system [92].

Monoamines also modulate the migration of GnRH neurons. As shown on the model of serotonin deficiency in rat fetuses, this monoamine stimulates their proliferation and further migration to the forebrain [13]. According to our data, suppression of catecholamine synthesis in rat embryos by α-methyl-p-tyrosine (αMPT, a competitive tyrosine hydroxylase inhibitor) beginning from ED11 leads to an increase in the number of GnRH neurons in the rostral segments of their migration route by ED17 and their accumulation in the zone of their entry into the forebrain by days 18–21 [93]. In addition to GnRH, monoamines also affect the thymic development and functioning of the immune system [94,95].

### 2.5. Transcription Factors

The initial stages of the differentiation of olfactory placodes and the precursors of GnRH neurons are controlled by transcription factors (Table 1).

## 3. Development of the GnRH System in Different Pathological States

Disorders in the development of the GnRH system lead to impaired puberty and fertility in adults. The reasons for the development of reproductive system disorders in most patients are still not fully determined. Genetic mutations in genes that determine the synthesis of factors involved in the migration of GnRH neurons are detected in no more than 50% of patients [115]. Despite the fact that the proportion of certain genetic causes of underdevelopment of the reproductive system is growing, adverse factors affecting the developing human body in early ontogenesis can significantly influence the development of the GnRH system [37,50,116,117]. The best known disease associated with impaired migration of GnRH neurons is the so-called Kallmann syndrome. The diagnosis of Kallmann syndrome is based on the identification of impairments in sexual development: a decrease in the mass of the gonads, as well as low secretion of gonadotropins and sex steroids, and in addition, these symptoms are accompanied by a loss of smell (anosmia). When there is Kallmann syndrome in a human fetus with a genetic mutation in the X-linked gene *KAL1*, there is a complete impairment of the penetration of GnRH neurons into the brain, with neurons located in the nasal region along the dorsal surface of the ethmoid plate of the ethmoid bone [118]. Receptor, which is activated by the prokineticins (PRKR2), and the mouse nasal embryonic GnRH factor gene (NELF) are required for olfactory axonal outgrowth and GnRH neuronal migration in mice [119].

Reproductive impairment caused by decreased GnRH secretion is not always associated with impaired neuronal migration. Abnormal development of the gonads without defects in the development of the olfactory system in humans is called idiopathic hypogonadotropic hypogonadism. This pathology is more common in men and manifests itself in the early postnatal period [120]. The absence of puberty against the background of a decrease in GnRH secretion was also found in women [120].

For studies of pathological processes developing in the reproductive system, hypogonadal mice (hpg mice) are widely used. These mice have a general underdevelopment of the gonads and disorders of the reproductive system. They have a spontaneous deletion mutation of 33.5 kilobases encompassing the distal half of the GNRH1 gene, which completely disrupts the transcription and synthesis of GnRH, leading to a lifelong deficiency of gonadotropins and sex steroids. In male *hpg* mice, changes in the concentration of amyloid precursors, as well as a decrease in the level of IL-1β in the hippocampus and choline acetyltransferase per neuron, were revealed, similar to changes in patients with Alzheimer’s disease [121].

Currently, more and more clinical and experimental data are coming in on the negative influence of various unfavorable factors on the developing fetus, including the HPG axis. Their impact leads to persistent changes in the epigenetic regulation of gene transcription, and as a consequence, to phenotypic changes [116,122,123]. The HPG axis disorders are based on epigenetic modification of the estrogen receptor (ERα) gene promoter and subsequent changes in the expression of this gene [124,125]. It has been shown that estrogens increase the expression of the KISS1 gene in the brain of mice by the acetylation of histones in its promoter region in the anteroventral periventricular nucleus of the hypothalamus and suppress its expression by the deacetylation of histones in the arcuate nucleus. Epigenetic regulation of the KISS1 gene by estrogen-positive feedback induces the release of GnRH [126]. Suppression of KISS1 gene expression in female rats in the medial preoptic region of the hypothalamus, induced by systemic inflammation in the neonatal period, significantly slows down the onset of puberty [127].

One of the risk factors is a bacterial infection that induces inflammation in both the mother and the fetus [37,47,50,51,52,128]. Bacterial infections, particularly asymptomatic infections during pregnancy that are not normally treated, can lead to serious complications including pre-term parturition, low birth weight, and CNS damage [51,129,130,131,132].

Experimental studies often use LPS, a major component of the outer membrane in bacteria. LPS is a strong inducer of innate immunity consisting primarily of cytokine induction, inflammation, fever, complement cascade activation, hypothalamic–pituitary–adrenal (HPA) axis activation, and sickness behavior [47,51]. By inducing an immune response in the mother, LPS is able to alter the level of cytokines in the fetus. Proinflammatory cytokines are probably a link between maternal infection and subsequent disruptions in the development and further functioning of various brain systems in the offspring.

According to our data, activation of the immune system of rats or mice by LPS (*Escherichia coli*) on the 12th day of pregnancy leads to a decrease in the rate of migration of GnRH neurons from the nasal region to the brain and is accompanied by an increase in the level of IL-6, LIF, and MCP-1 in the blood of the mother and the fetuses [37,117]. In sexually mature offspring, there is a decrease in the level of GnRH in the hypothalamus and a reduction in the level of gonadotropins and sex steroids in the peripheral blood [50]. The effect of immunological stress on the development of the fetal GnRH system depends on the period of exposure. If such activation takes place at the initial stage of GnRH neuronal migration in the fetus, it results in the overall disorganization of this process. At the same time, LPS administration to pregnant females at subsequent stages of neuronal migration does not lead to a redistribution of GnRH neurons in the fetal brain, which indicates that some other mechanisms become involved in the regulation of their migration [37].

The appearance of GnRH neurons in the forebrain with a delay, apparently, causes changes in the formation of the necessary axonal connections, which leads to impairments at key points in the development of the HPG axis. In adult animals, LPS-induced inflammation has been proposed to suppress axonal transport of GnRH mRNA to GnRH neurons in the preoptic region and the median eminence of the hypothalamus, where their terminals are projected and GnRH mRNA is detected [133]. The formation of a synaptic network that modulates the function of GnRH neurons is controlled by the integrative activity of internal and external signals. Thus, suppression of GnRH synthesis in the hypothalamus and suppression of gonadotropin secretion in the pituitary gland after systemic administration of LPS to rats are accompanied by an increase in IL-1β secretion and tumor necrosis factor (TNF) α in the medial preoptic region of the hypothalamus [134]. It is known that an increased level of TNFα in the blood induces sepsis and in the brain, it causes apoptosis of developing oligodendrocytes, mediated by the apoptosis-inducing factor (AIF), which is translocated into the nucleus under the influence of TNFα [135,136]. An LPS-induced intrauterine inflammation in mice, accompanied by increased levels of TNFα, leads to hypomyelination and diffuse brain damage, both in the white matter and in the gray matter of fetuses, including the hypothalamus, the thalamus, and the hippocampus [42,137].

With the central administration of IL-1β, an increase in the levels of β-endorphin and tachykinins is observed. They are involved in the retransmission of the cytokine signal into GnRH neurons and suppress their functioning [138]. IL-1α and granulocyte macrophage colony-stimulating factor (GM-CSF) block NO-induced GnRH secretion in the mediobasal region of the hypothalamus, which in turn blocks the pulsatile secretion of LH into the blood and suppresses GnRH-regulated sexual behavior [59]. Through GnRH neurons, various neurotransmitters and neuropeptides, such as monoamines, GABA, neuropeptide Y, opioids, cytokines, KISS1, as well as leptin, transmit external stimulus signals that affect the state of the HPG system [2,18,23,82,92,133].

An LPS-induced inflammation in the mother during pregnancy causes impairments in the formation of GABA-, dopamine- and serotonin-producing neurons in the developing fetal brain [43,132]. Administration of LPS to female rats on the 11th day of gestation leads to a decrease in the number of dopaminergic neurons and an increase in the activity of microglia, as well as to an increase in the level of proinflammatory cytokines, mainly TNFα, in the substantia nigra in postnatal offspring [43]. Moreover, LPS can influence the differentiation of monoaminergic neurons not only in the brain stem, but also in other brain structures, including the hypothalamus in fetuses. After the introduction of LPS, the following was observed: a decrease in the expression of the enzyme synthesizing dopamine (tyrosine hydroxylase) in the substantia nigra and of the enzyme synthesizing serotonin (tryptophan hydroxylase) in the dorsal raphe nucleus, a decrease in the levels of dopamine and serotonin in the olfactory bulbs of the frontal cortex, the nucleus accumbens, the striatum, the amygdala, the hippocampus, and the hypothalamus, as well as a decrease in the expression of the enzyme synthesizing GABA and reelin in the dentate gyrus and the CA1 in offspring [43,139,140].

Thus, activation of the immune system in early ontogenesis triggers a cascade of intermediators that cause impairments in the formation of both the immune system and the HPG system, which leads to an increased risk of immunological, behavioral, and reproductive disorders in offspring.

## 4. Effects of GnRH on the Development and Functioning of the Immune System

GnRH is an important signaling molecule in neuroendocrine-immune interactions [2,141]. Hypothalamic and extracerebral GnRHs are involved in the regulation of the development and functioning of the immune system at different stages of ontogenesis. At the same time, the level of GnRH in the general circulation in adults is low, while in fetuses its level is much higher than that in postnatal life [11,12]. After removal of the hypothalamus by in utero encephalectomy in 18-day-old rat fetuses, the level of GnRH in the thymus and peripheral blood of 21-day-old fetuses of both sexes is halved compared to the norm [11]. Since in the prenatal period of development, the blood–brain barrier in mammals is just beginning to form and is poorly functioning, at least half of the GnRH enters the periphery from the fetal brain.

According to current conceptions, hormones that control certain functions in adults act on the formation of these functions in perinatal ontogenesis [9,10,142]. They control the growth and differentiation of various fetal tissues, including lymphoid tissue. Changes in the physiological concentrations of hormones, including GnRH, as well as the effects of various unfavorable environmental factors on the developing fetus, cause impairments in the programming of the regulatory mechanisms of the neuroendocrine and immune systems [143]. As a result, disorders occur in the immune system, primarily in the development of the central organs (the thymus and bone marrow), which subsequently leads to impairments in the functioning of peripheral lymphoid organs (the spleen and lymph nodes) [6].

**Figure 3:**
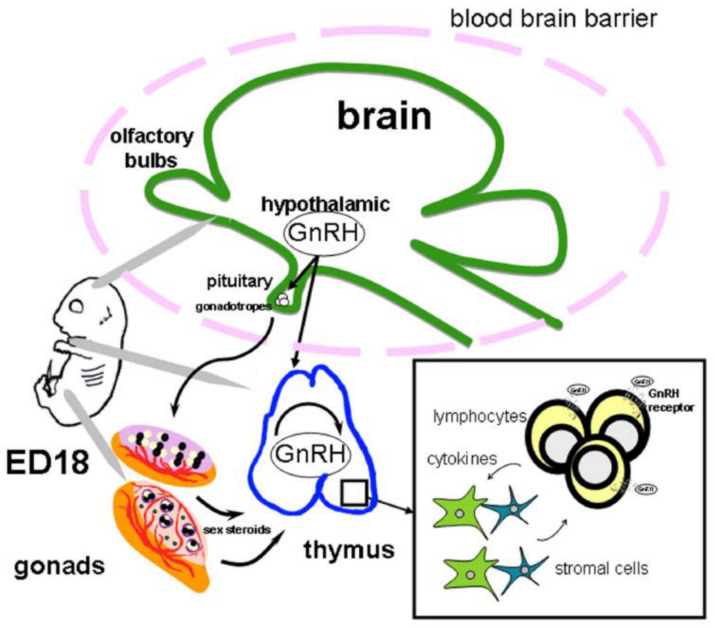
Model of the gonadotropin-releasing hormone (GnRH) regulation in the thymus during ontogeny. During prenatal development, before the establishment of the hypothalamic-pituitary-gonadal regulation in rodents, hypothalamic GnRH is found in the general blood circulation in high concentrations and can provide a direct effect on the morphogenesis of thymus via specific receptor in thymocytes. In addition, the effect of GnRH synthesized in thymus can be realized by autocrine or paracrine regulation. During early development, the effect of GnRH is mediated by cytokines whose synthesis in the thymus is upregulated by this neuropeptide. During the postnatal period, GnRH is involved in the bidirectional programming of the both neuroendocrine and immune functions via gonadotropins and sex steroids. Sex steroids modulate the GnRH level in the thymus. Abbreviation: ED—embryonic day.

GnRH is involved in the regulation of the development and functioning of thymic T lymphocytes already in prenatal ontogenesis. Central and peripheral blockade of GnRHRs by a selective antagonist or specific antibodies in rat fetuses leads to suppression of the mitogen-induced proliferative response of fetal thymocytes, while administration of GnRH completely restores the immune response to normal [11,144]. In utero blockade of GnRHRs at the peak of their expression in the thymus of rats on ED17 by the antagonist causes suppression of the mitogen-induced proliferative response of T-lymphocytes not only in fetuses, but also in mature offspring. After the administration of the GnRH antagonist to sexually mature rats, the impairments in the development of the cellular immune response were short-lived and reversible [6]. Blockade of GnRHRs by an antagonist in rats for five neonatal days subsequently leads to suppression of the humoral and cellular immune response [28], at the same time, a single administration of the antagonist to rat pups on the 3rd day after birth does not affect the functional activity of thymocytes in adults [6]. After neonatal chronic exposure to the antagonist, a decrease in the mass of the thymus and the number of mature T- and B-lymphocytes in lymphoid organs and peripheral blood was observed in sexually mature rats and primates [145]. Differences in the effects of GnRH on the immune system at different periods of ontogenesis may be associated with the involvement of different regulatory mechanisms. In rat fetuses, hypothalamic control of gonadotropin secretion by the pituitary gland is formed on ED21. At the same time, axonal pathways of GnRH transport to the portal circulation are formed and expression of GnRHR on gonadotropocytes begins, which is most pronounced by 10-12 days after birth [12,146]. The high level of GnRHR expression on ED17-18, as well as the absence of long-term consequences of a single administration of the GnRH antagonist at the very beginning of the neonatal period, indicates the regulatory effect of GnRH on thymocyte formation at the end of the second decade of intrauterine development. With chronic exposure to the antagonist in the neonatal period, the effects of GnRH are probably realized through gonadotropins. The schematic representation of GnRH effects on the thymus development in fetal rats is presented on Figure 3.

In the prepubertal period, GnRH modulates the development of hematopoietic stem cells in the central organs of the immune system. Two to three weeks after treating female mice during this period with a long-acting analog of GnRH lupron depot (prepared as a long-term slow release formulation), the number of T-lymphocytes in the thymus and B-lymphocytes in the bone marrow is significantly reduced. This leads to a decrease in their number and suppression of cellular immunity in the peripheral lymphoid organs [147]. GnRH and its agonists prevent cell apoptosis [148], normalize the number of T-lymphocytes in the thymus, mainly CD4 T helper cells, and their functional activity, both in pre- and postpubertal periods [6,149].

The mechanisms of GnRH action on the immune system are not fully understood, and they seem to be different at different stages of ontogenesis. One of the possible mechanisms for regulating the number of lymphocytes is the ability of GnRH to induce the expression of cytokines and their receptors. It is known that cytokines are involved in the processes of proliferation and differentiation of lymphocytes, in the regulation of cell-cell interactions, apoptosis, and morphogenesis [150,151]. Moreover, in different periods of ontogenesis, they have different effects. In adults, cytokines are involved in the regulation of the immune response to various foreign antigens, while in early development they model the processes of formation of various organs and tissues of the fetus, including brain structures [87,152].

In the postnatal period of life, GnRH enhances the expression of the IL-2 receptor (IL-2Rγ) on T-lymphocytes: this receptor triggers an IL-2 dependent mitogen-induced proliferative response [153,154]. In culture of B-lymphoblastoid cells, GnRH also enhances their proliferative activity, which is most pronounced in the presence of IL-2 [155].

In prenatal ontogenesis, GnRH in physiological concentrations does not induce the synthesis of IL-2 in the fetal thymus, while the synthesis of IL-4, IL-10, TNFα and interferon (IFN) γ increases. GnRH has the greatest effect on the synthesis of the lymphocyte differentiation factor IL-4, which, as is known, in synergy with IL-10, regulates the synthesis of other cytokines [6,156]. The GnRH antagonist suppresses the expression of IL-4, IFNγ, IL-1β, and TNFα, while IL-1α, IL-2, and IL-10 remain unchanged. It was shown that in the fetal thymus of mice, the synthesis of IL-1β, IL-4, IL-5, IL-6, IL-7, IFNγ, and TNFβ begins from ED14, while that of IL-1α, IL-2, and IL-3 starts from embryonic day 16 [156]. At the same time, the synthesis of IL-7, which stimulates the proliferation, but not the differentiation of T-lymphocytes, slows down by the time of the synthesis of IL-2, which indicates the involvement of certain cytokines at certain stages of thymus development. Thus, in prenatal ontogenesis, GnRH is probably, like many hormones and mediators, an inducer of development, exerting a morphogenetic effect conditioned by cytokines on the central organs of the immune system, in particular the thymus.

Sex steroids, and first of all testosterone, the synthesis of which by Leydig cells in rat fetuses was detected already on ED 18-19 [157], modulate the molecular processing of the GnRH precursor and, therefore, the GnRH level in the hypothalamus and thymus [158]. In the thymus of castrated 2-month-old male rats, the level of the GnRH precursor decreases, while the GnRH level increases in the thymus and decreases in the hypothalamus. Testosterone replacement therapy prevents these effects [158]. At the same time, the level of GnRH mRNA in castrated rats does not change, which indicates the post-translational effect of testosterone, which suppresses GnRH processing. Since in non-castrated males the concentration of GnRH in the thymus was lower than in the hypothalamus, while the level of the precursor of GnRH in the thymus was much higher, the authors suggested that the regulation of molecular processing by testosterone in these tissues occurs in different ways.

According to Jacobson and Ansari (2004) [159], the stimulating effects of estrogens and the suppressive effects of androgens on the number and functioning of B-lymphocytes are realized only in the presence of GnRH. Along with the modulation of the immune response, the participation of GnRH and sex steroids in autoimmune processes is assumed [35,160]. After treating systemic lupus erythematosus-prone male and female mice, who were F_1_ hybrid (SWR × NZB), with GnRH antagonist, a decrease in the total levels of immunoglobulin (IgG) and anti-DNA antibodies, a decrease in the severity of clinical manifestations of the disease, and an increase in life expectancy were observed in both sexes. At the same time, after the introduction of the GnRH agonist (native decapeptide) or its analogs, exacerbation of the disease was observed in females, but not in males [160]. The observed effects in the course of autoimmune diseases, as suggested by the authors, may be due to sex differences in the expression of GnRHR or G proteins. After 2 weeks of treating mice with systemic lupus erythematosus with GnRH, the expression of GnRHR and the binding of its ligand in the spleen of ovariectomized but not castrated animals are suppressed. At the same time, an increase in the expression of IL-2 and its receptor is observed in females. GnRH realizes its effects through a G protein-coupled receptor, specifically through two highly homologous G proteins Gαq and Gα_11_ (Gaq/_11_). It turned out that in the spleen of females, the expression of Gaq/_11_ is more pronounced than in males, and their expression is enhanced by GnRH in both intact and ovariectomized mice [161].

Thus, GnRH can control the formation and functioning of the immune system through the HPG axis, as well as carry out autocrine or paracrine regulation of the immune response. At the same time, adverse effects in the perinatal period, as a rule, might cause irreversible or long-term current structural and functional disorders, while in puberty, these disorders are short-lived and reversible.

## 5. Approaches to Preventing or Reversing Disorders Induced by Acute Inflammation in Early Ontogenesis

The increase in the number of couples of childbearing age suffering from infertility is becoming a serious problem for men and women and constitutes 8–15% worldwide. Genetic, physiological, social, and environmental factors contribute to the development of infertility, especially when exposed to them during critical periods of the formation of the HPG axis. In infertile men, various viruses are often detected in the semen and tissues of the reproductive tract [1]. A low sperm count is found in 15–20% of young men [162]. In women, asymptomatic diseases caused by gram-negative bacteria are common [163], which is especially dangerous during gestation. The consequences of negative effects on the fetus are not always traced; therefore, the therapy carried out at puberty, as a rule, does not allow the elimination of the identified fertility disorders.

Currently, data are accumulating on the possibility of preventing or reversing the short-term and long-term consequences of intrauterine inflammation. An effective neuroprotective agent for the fetus is magnesium sulfate (Mg), which is prescribed to women at risk of preterm birth [164]. Experimental studies have shown that Mg administered to female rats on ED16 before LPS injection significantly decreases brain neuronal nitric oxide synthase, nuclear factor NF-κB, and chemokine (C-C motif) ligand 2 protein, the levels of which increase after exposure to LPS. The Mg effect was mediated through the *N*-methyl-d-aspartate receptor (NMDAR). In addition, Mg reduced the increased levels of NO and proinflammatory cytokines in the fetal brain [165].

Antioxidant *N*-acetyl-cysteine (NAC) also prevents the rat fetal brain from inflammatory cytokine responses to LPS [166]. Suppression of the synthesis of TNFα, IL-6, and IL-10, as well as suppression of the development of local inflammation in the fetal brain, led to the reversal of the long-term negative consequences of inflammation [137]. In an in vitro model, it has been demonstrated that NAC inhibits an NF-κB activated pathway and subsequent phospholipid metabolism, proinflammatory cytokine release, and protease activity in human fetal membranes [167].

Studies of pregnant women with Zn deficiency have shown that they have increased risks of low birth weight and small for gestational age infants. At the same time, the levels of C-reactive protein, TNFα, and IL-8 in maternal serum, as well as the concentration of the nuclear factor NF-κB p65, significantly exceed the normal level [168]. According to Chua et al. (2012) [169], activation of the maternal immune system by LPS in mid- and late pregnancy induces the production of metallothionein, which redistributes Zn in the mother, limiting its entry into the fetus. Zn deficiency in the fetus leads to damage in development of the nervous system. Zn co-administered with LPS to mice on ED16 prevents astrogliosis, brain cell death, and increased TNFα synthesis.

Protective anti-inflammatory properties have also been found in polyunsaturated fatty acids (“n-3 PUFAs”, known as omega-3) [170] and vitamin D [171]. The intake of vitamin D by pregnant women results in an increase in the weight and height of the newborn [172]. It is known that the quality, quantity, and timing of food consumption during pregnancy affect the growth and development of the fetus [173]. Newborns with low birth weight, caused by improper nutrition of the mother, have an increased risk of mortality from congenital heart disease and are more likely to suffer from the development of metabolic syndromes, obstructive pulmonary diseases, renal failure, allergic diseases, as well as reproductive dysfunction in young offspring. The developing fetus is most sensitive to substances such as amino acids and folic acid (B9), as well as vitamins B6 (pyridoxine) and B12 [174]. Their increased levels in early ontogenesis can reprogram the development of systems, and therefore, the use of various additives, including vitamins, during pregnancy should be treated with caution.

We have demonstrated the possibility of reversing the structural and functional disorders of the HPG system in male and female rats induced by prenatal exposure to LPS. In the prepubertal period, males showed increased levels of estradiol, while females showed increased levels of testosterone. Antagonists of sex steroids, introduced during this period, normalized inflammation-induced disorders [51,52].

In recent years, fundamentally new anti-inflammatory “genetically engineered biological drugs” have been created, the use of which has made it possible to significantly increase the effectiveness of pharmacotherapy. These include monoclonal antibodies against certain determinants of immune cells or proinflammatory cytokines, as well as hybrid protein molecules that inhibit the activity of cytokines or the interaction of T- and B-lymphocytes. Tocilizumab (TCZ), which is clinically used mainly for the treatment of arthritis, may be a promising corrective drug that suppresses inflammation in the early stages of development [175]. TCZ is a recombinant humanized anti-IL-6 receptor monoclonal antibody of the IgG1_k_ subclass [176]. IL-6 is a key marker of neuroinflammation and lethal sepsis following bacterial or viral activation of the immune system. It has been proven experimentally that an increase in the level of IL-6 in female mice in early pregnancy causes behavioral and transcriptional changes in the brain of adult offspring. Antibodies against IL-6 (IgG1), administered to pregnant mice immediately after their activation by double-stranded RNA (poly (I:C)), normalize the induced changes in the adult offspring [177].

Attempts are being made to neutralize increased levels of proinflammatory cytokines by an intravenous administration of human IgG (IVIG) [178,179,180]. In experimental rodent models with LPS-induced endotoxemia, IgG and IgM suppressed increased cytokine synthesis and the development of immunosuppression [181]. According to our preliminary data, IgG administered intravenously to pregnant female mice 40 min after LPS administration reverses, although not completely, structural abnormalities of the gonads in male offspring.

The mechanism of action of Ig during bacterial infection is not yet fully understood. It is assumed that Ig binds to Fc-γ receptors on macrophages [182], decreasing their sensitivity to signals through Toll receptors, and therefore to LPS, which leads to a decrease in the synthesis of proinflammatory cytokines and protection from LPS-induced death in animals [178,183,184]. Ig is able to enhance the expression of negative regulators of LPS-induced NF-kB signaling. IgG induces the activation of JNK (Jun kinase) and the synthesis of the signal peptide activin A in anti-inflammatory macrophages, causing their generation, as well as inhibiting the granulocyte-macrophage colony-stimulating factor (GM-CSF)-initiated activation of the transcription factor STAT5 (signal transducer and activator of transcription-5), limiting the differentiation of proinflammatory macrophages [178]. Moreover, activin A makes a significant contribution to the effects of IgG. An increase in the level of activin A in the blood after IVIg administration contributes to the beneficial effect of Ig on women with recurrent reproductive failure.

To preserve reproductive health at childbearing age in men and women, we focus on the possibility of correcting the effects of bacterial infection in the mother in early pregnancy (first trimester in humans). Two critical periods can be defined: 1. in the prenatal period, the administration of IgG or antibodies to proinflammatory cytokines at the initial stages of their synthesis; 2. in the neonatal and early infantile periods, the administration of sex steroid antagonists, if there are elevated levels of sex steroids in the blood [47].

## 6. Conclusions

An analysis of existing research and our own data have shown that the regulation of the formation and functioning of the HPG and immune systems in a developing fetus is carried out with a close interaction of these systems. Epigenetic mechanisms are implemented during perinatal ontogenesis that ensures the adaptive plasticity of the physiological systems. Impairments in the molecular mechanisms of regulation in critical periods of development can cause long-term or irreversible changes in the functioning of the HPG and immune systems. Their reprogramming with various stressful stimuli during this period can lead to the emergence of metabolic, immunological, and reproductive disorders in the offspring. However, regulatory epigenetic mechanisms of development are not strictly genetically controlled. These processes are characterized by a high sensitivity to various regulatory factors, which makes it possible to correct disorders. 

## Figures and Tables

**Table 1:** The most general transcriptional factors participating in organization of GnRH neuron development and its migratory route formation.

Transcriptional Factor	General Action	Action on GnRH	References
Pax-6,7	The role of Pax-6 in eye and nasal development	Its expression appears in the olfactory ensheathing cells and vomeronasal organ. PAX6 and AP-2 define distinct progenitor cells mixed within the developing nasal placode	[96,97]
Eya-1,2	The role in developmental process conserved across vertebrates	It is initially expressed in a row of eight cells located immediately anterior to the neural plate	[98,99]
Six-1,3,6	Six is restricted to the developing eye and brain	Mice lacking Six1 (Six1−/−) exhibited defective early neurogenesis in the olfactory epithelium. Loss of Six6 expression disrupts male fertility because of decreased follicle-stimulating hormone release	[99,100,101]
OTX-1,2	Involvement in the early development of CNS	The role in the initiation and guidance of directed migration. In mice with deletion of the transcription factor Otx2 found fewer GnRH neurons	[102,103]
Gli3	It is necessary for the development of olfactory system	Regulates vomeronasal neurogenesis, olfactory ensheathing cell formation and GnRH neuronal migration	[104,105]
Ebf2	Involvement in numerous developmental processes, ranging from B-cell development to neuronal differentiation	It is expressed in migrating GnRH neurons on embryonic day 11 (ED11). Mice with disrupted expression of Ebf2 retained GnRH neurons clustered in the nasal mesenchyme	[106,107]
Nhlh2	Its expression in regions of the hypothalamus as well as the pituitary	Mice with disrupted expression of Nhlh2 had a loss of GnRH neurons in adulthood that occurred sometime between birth and adulthood (loss of 60% in females and 30% in males)	[108,109]
VAX1	It is essential for the formation of the eye, ventral forebrain and palate	GnRH staining in Vax1null mice show a total absence of GnRH expression in the adult. Using the immortalized model GnRH neuron cell lines, GN11 and GT1-7, it was show that VAX1 is a direct regulator of GnRH transcription by binding key ATTA sites within the GnRH promoter.	[110,111]
Mash-1, Math4A, Math4/neurogenin1, NeuroD	Involvement in gliogenesis and neurogenesis	Differentiation into the olfactory epithelium	[101,112]
Olf-1, GATA-4	Involvement in brain development	Its expression in the olfactory epithelium Its expression is necessary for GnRH pulse activity	[113,114]
AP-2α	Involvement in craniofacial morphogenesis	Its expression only in the respiratory epithelium. It prevents recapitulation of developmental programs within the respiratory epithelium that lead to expression of GnRH and peripherin phenotypes	[113]

## Abbreviations

CNS	Central nervous system
ED	Embryonic day
ERα	Estrogen receptor alfa
FSH	Follicle-stimulating hormone
GABA	Gamma-aminobutyric acid
GM-CSF	Granulocyte macrophage colony-stimulating factor
GnRH	Gonadotropin-releasing hormone
HGF/SF	Hepatocyte growth factor/scatter factor
HPG	Hypothalamic-pituitary-gonadal (system)
IFNγ	Interferon gamma
IgG	Immunoglobulin G
IL	Interleukin
KISS1	Kisspeptin
LH	Luteinizing hormone
LIF	Leukemia inhibitory factor
LPS	Lipopolysaccharide
MCP-1	Monocyte chemoattractant protein-1
SDF-1	Stromal cell-derived factor 1 and one of its receptors **CXCR4**
SEMA	Semaphorin
TNF	Tumor necrosis factor (alpha and beta)
